# A comprehensive model of maternal health behavior: the interplay of sociodemographic and digital determinants in Nigeria

**DOI:** 10.1590/0102-311XEN184125

**Published:** 2026-09-18

**Authors:** Muyiwa Oladosun, Anthony Etim

**Affiliations:** 1 Covenant University, Ota, Nigeria

**Keywords:** Antenatal Care, Maternal Behavior, Maternal Health, Obstetric Delivery, Cuidado Pré-Natal, Comportamento Materno, Saúde Materna, Parto Obstétrico, Atención Prenatal, Conducta Materna, Salud Materna, Parto Obstétrico

## Abstract

This study develops an integrated model to identify significant sociodemographic and digital determinants of maternal health service utilization among married women in Nigeria. A cross-sectional analysis of data from the 2018 *Nigeria Demographic and Health Survey* (NDHS) was conducted on 28,888 married women aged 15-49. All analyses were conducted using the Complex Samples module in IBM SPSS Statistics, applying design-corrected variance estimation appropriate for the stratified cluster design of the NDHS. The design-weighted prevalence of six or more antenatal care visits was 25%, and the prevalence of facility-based delivery was 28.7%. A comprehensive multivariate binary logistic regression model assessed influences on both outcomes. Higher education and household wealth were the strongest determinants. Mobile phone ownership was consistently and positively associated with both outcomes (OR = 1.29, 95%CI: 1.16-1.43 for antenatal care; OR = 1.36, 95%CI: 1.24-1.49 for facility delivery). Internet use was independently and positively associated with antenatal care attendance, while frequent internet use was independently and negatively associated with antenatal care attendance. Mobile financial transactions were associated with significantly lower odds of facility delivery. A complex interplay of structural inequities and digital influences shapes maternal health behavior in Nigeria. Policies must be multisectoral, addressing foundational determinants such as education and wealth, while recognizing that the impact of digital technology on health choices is not uniformly linear.

## Abstract (pt)

Este estudo desenvolveu um modelo integrado para identificar os determinantes sociodemográficos e digitais significativos à utilização dos serviços de saúde materna entre mulheres casadas na Nigéria. Uma análise transversal dos dados da *Pesquisa Demográfica e de Saúde da Nigéria* (NDHS, acrônimo em inglês) de 2018 foi realizada com 28.888 mulheres casadas entre 15 e 49 anos. Todas as análises foram realizadas usando o módulo Amostras Complexas em IBM SPSS Statistics, aplicando uma estimativa de variância corrigida pelo projeto apropriada para o projeto de cluster estratificado da NDHS. A prevalência ponderada pelo desenho de seis ou mais visitas de cuidados pré-natais foi de 25%. A prevalência de parto em instalações foi de 28,7%. Um modelo abrangente de regressão logística binária multivariada avaliou as influências em ambos os desfechos. Ensino Superior e a riqueza familiar foram os determinantes mais fortes. A posse de celular esteve consistente e positivamente associada a ambos os desfechos (OR = 1,29, IC95%: 1,16-1,43 para cuidados pré-natais; OR = 1,36, IC95%: 1,24-1,49 para partos em unidades de saúde). O uso da internet esteve associado independente e positivamente à frequência de cuidados pré-natais, enquanto o uso frequente da internet esteve independente e negativamente associado à frequência de cuidados pré-natais. Transações financeiras por celular foram associadas a chances significativamente menores de partos em unidades de saúde. Uma complexa interação entre desigualdades estruturais e influências digitais molda o comportamento da saúde materna na Nigéria. Políticas devem ser multisetoriais, abordando determinantes fundamentais como educação e riqueza já que o impacto da tecnologia digital nas escolhas de saúde não é uniformemente linear.

## Abstract (es)

Este estudio desarrolló un modelo integrado para identificar determinantes sociodemográficos y digitales significativos para el uso de los servicios de salud materna entre las mujeres casadas en Nigeria. Se realizó un análisis transversal de los datos de la *Encuesta Demográfica y de Salud de Nigeria* (NDHS, por su sigla en Inglés) de 2018 con 28.888 mujeres casadas de entre 15 y 49 años. Todos los análisis se realizaron utilizando el módulo Muestras Complejas en IBM SPSS Statistics, aplicando una estimación de varianza corregida por el diseño, adecuada para el diseño estratificado por conglomerados de la NDHS. La prevalencia ponderada por el diseño de seis o más visitas de atención prenatal fue del 25%. La prevalencia de parto institucional fue del 28,7%. Se utilizó un modelo integral de regresión logística binaria multivariada para evaluar las influencias en ambos resultados. La enseñanza superior y la riqueza familiar fueron los determinantes más fuertes. La posesión de teléfono móvil se asoció de manera consistente y positiva con ambos resultados (OR = 1,29, IC95%: 1,16-1,43 para la atención prenatal; OR = 1,36, IC95%: 1,24-1,49 para el parto institucional). El uso de internet se asoció de forma independiente y positiva con la frecuencia de atención prenatal, mientras que el uso frecuente de Internet se asoció de forma independiente y negativa con la frecuencia de atención prenatal. Las transacciones financieras realizadas a través del teléfono móvil se asociaron con probabilidades significativamente menores de partos institucionales. En Nigeria, una compleja interacción entre las desigualdades estructurales y las influencias digitales moldea el comportamiento de la salud materna. Las políticas deben ser multisectoriales y abordar determinantes clave como la educación y la riqueza, puesto que el impacto de la tecnología digital en las decisiones de salud no es uniformemente lineal.

## Introduction

The imperative to improve maternal health outcomes in Nigeria is intrinsically linked to promoting positive health behaviors, such as consistent attendance at antenatal care and choosing to deliver in a health facility. These actions are critical for reducing the country’s high rates of maternal and neonatal mortality ^1^ ^,^ ^2^ ^,^ ^3^. While the drivers of these behaviors are complex, the advent of digital technology has introduced a new and powerful dimension to public health interventions. Mobile health (mHealth) tools, from simple SMS reminders to more complex applications, are increasingly recognized as a vital means of encouraging proactive health-seeking behavior by delivering timely information and support directly to women ^4^ ^,^ ^5^. However, digital tools do not operate in a vacuum. The enduring influence of traditional sociodemographic realities shapes their effectiveness. A woman’s decision to seek care is a product of both her digital access and her socioeconomic context, including her education, wealth, and geographic location ^6^ ^,^ ^7^ ^,^ ^8^ ^,^ ^9^. While many studies have examined these factors in isolation, there is a need for a comprehensive, integrated model that assesses the simultaneous influence of both traditional and digital determinants on maternal health actions at a national scale. This study is motivated by the need to develop such a model and provide policymakers with a comprehensive picture.

This study is underpinned by a multi-layered theoretical approach that integrates structural, technological, and psychological perspectives to explain health behavior. The Social Determinants of Health framework provides the overarching context, positing that structural conditions, such as income, education, and geography, are the fundamental drivers of health outcomes and behaviors ^10^ ^,^ ^11^. This is complemented by the health belief model (HBM), which explains the psychological mechanisms of action. The HBM suggests that a woman is more likely to adopt a health behavior if she perceives a health threat and believes in the efficacy of the recommended action, particularly when prompted by “cues to action,” such as an SMS reminder for an antenatal care visit ^12^ ^,^ ^13^. The technology acceptance model (TAM) further explains why a woman would engage with such digital cues in the first place, based on the perceived usefulness and ease of use of the technology ^14^ ^,^ ^15^ ^,^ ^16^. Finally, Intersectionality Theory provides a critical lens to understand how these factors combine, recognizing that a woman’s overlapping identities create compounded barriers that a single-factor analysis would miss ^17^ ^,^ ^18^.

The body of research on maternal health behavior in Nigeria is extensive. A substantial body of evidence has firmly established the significant role of sociodemographic and economic factors. Studies consistently demonstrate that women with higher levels of education, greater household wealth, and urban residence are significantly more likely to attend the recommended number of antenatal care visits and deliver their babies in a health facility ^1^ ^,^ ^5^. Education, in particular, is a powerful enabler, as it enhances a woman’s health literacy, decision-making autonomy, and ability to navigate complex health systems ^1^. Similarly, household wealth mitigates the direct and indirect costs associated with accessing care ^7^ ^,^ ^19^. The urban-rural divide is another critical determinant ^5^. These structural factors are often compounded by cultural and social norms, including a partner’s education level and women’s decision-making power within the household ^6^ ^,^ ^20^ ^,^ ^21^. 

Concurrently, a growing field of evidence highlights the positive impact of digital interventions. Systematic reviews have shown that mHealth tools, particularly SMS-based interventions, can effectively increase antenatal care attendance and skilled birth attendance in low- and middle-income countries ^4^ ^,^ ^5^. In Nigeria, numerous targeted projects have confirmed this potential. For instance, a study in Cross River State found that a simple text-messaging intervention dramatically increased the rate of facility-based deliveries among participants ^22^. Similarly, research has shown that mobile messaging can significantly improve the uptake of formal maternal health services ^23^. More advanced mobile applications are also emerging as powerful tools ^24^.

Despite the strength of these two bodies of literature, a significant gap remains. Most studies tend to focus either on traditional sociodemographic determinants or on the impact of a specific digital intervention. However, they rarely integrate both into a single, comprehensive model at a national scale. Research on socioeconomic factors often includes digital access as a single variable without exploring the nuances of different types of digital engagement ^5^. Conversely, studies on mHealth interventions are frequently localized and may not be generalizable ^25^. Consequently, there is a scarcity of nationally representative research that simultaneously assesses the relative and combined influence of a full suite of background factors and various digital engagement indicators on ultimate behavioral outcomes. This study aims to fill this gap by constructing and testing such an integrated model.

This study addresses the central research question: What is the combined and independent influence of a comprehensive set of sociodemographic and digital factors on the maternal health behaviors of married women in Nigeria? This study hypothesizes that while traditional determinants are powerful, digital factors have a significant and independent effect, though their influence may be complex and nonlinear. The primary objective is to develop and test a comprehensive, integrated model of maternal health service utilization using nationally representative data from the 2018 *Nigeria Demographic and Health Survey* (NDHS).

## Methods

This study employed a quantitative, cross-sectional research design and conducted a secondary analysis of data from the 2018 NDHS. The NDHS is a nationally representative survey that provides robust, high-quality data on a wide range of demographic, socioeconomic, and health indicators across Nigeria. The study was conducted in the Federal Republic of Nigeria, a nation characterized by significant regional, cultural, and socioeconomic diversity.

The study population comprised married women of reproductive age (15-49 years) who were residents of Nigeria at the time of the 2018 NDHS data collection. The 2018 NDHS successfully interviewed a total of 41,821 women aged 15-49. For this analysis, the sample was filtered to include only women who reported being currently married or living with a partner. This yielded a final analytical sample of 28,888 women.

The focus on women in marital or cohabiting unions was a deliberate methodological decision to ensure the analytical coherence of the model. The sociocultural and economic context of marriage in Nigeria significantly influences health-seeking behavior in ways distinct from those affecting unmarried women ^26^ ^,^ ^27^. For married women, maternal health choices are often not made autonomously; they are frequently collective household decisions, heavily influenced by the partner’s characteristics and the woman’s decision-making power within that specific relationship ^28^.

The 2018 NDHS was implemented by Nigeria’s National Population Commission (NPC) with technical assistance from ICF International. The survey utilized a stratified, two-stage cluster sampling design to ensure the sample was nationally representative. In the first stage, 1,400 Enumeration Areas (EAs) were selected from the 2006 *National Population and Housing Census* frame using a probability proportional to size method. In the second stage, a fixed number of 30 households were systematically selected from each of the chosen EAs following a household listing update. Data were collected by highly trained field staff using Computer-Assisted Personal Interviewing (CAPI).

The two primary health behavior outcomes for this study were operationalized as binary variables: Use of antenatal care (ANC), coded as 2 if the woman attended six or more antenatal care visits for her most recent birth, and 1 otherwise; and place of delivery, coded as 2 if the woman delivered her most recent baby in a health facility, and 1 otherwise. Explanatory variables were categorized into two groups: Sociodemographic (Background) Factors and Digital (Contextual) Factors. The sociodemographic variables included education, wealth quintile, residence, region, religion, number of household members, employment status, occupation, and the partner’s characteristics. Crucially, to avoid masking age-specific effects within the 15-49 cohort, the woman’s age variable was stratified into three distinct groups (29 or younger, 30-39, and 40 or older). The Digital (Contextual) Factors included mobile phone ownership, internet use, frequency of internet use, and use of a mobile phone for financial transactions.

All data management and statistical analyses were conducted using IBM SPSS Statistics (https://www.ibm.com/). To account for the stratified, clustered, and weighted nature of the 2018 NDHS, all inferential analyses were conducted using the Complex Samples module ^29^. An analysis plan file was created, specifying the first-stage stratification variable (V022), the primary sampling unit (V021), and the normalized sampling weight (V005 divided by 1,000,000) as the analysis weight, using with-replacement (WR) Taylor-series linearization as the variance estimator. The married women domain was defined using a binary indicator variable (MARRIED = 1) applied as the subpopulation, retaining all cases for correct variance estimation. The analysis began with design-weighted frequency distributions to produce Tables 1 and 2. Bivariate associations were examined using design-weighted cross-tabulations with row percentages, showing the proportion of each predictor category achieving each outcome. Associations are reported as design-weighted row percentages, as a formal independence test could not be computed under the current configuration because the domain variable could not be specified as a first-stage stratification variable. The core of the analysis was a comprehensive multivariate binary logistic regression using simultaneous entry of all sociodemographic and digital explanatory variables to assess their independent effects. Odds ratios (OR) with 95% confidence intervals (95%CI) were obtained, with code 1 specified as the reference category for each predictor. The F-adjusted Rao-Scott test was used. A p-value of less than 0.05 was used as the threshold for statistical significance. Model fit was assessed using the classification table, as the Hosmer-Lemeshow test and Nagelkerke R-squared are not available for design-corrected complex samples logistic regression. Multicollinearity diagnostics were performed using standard OLS regression prior to the logistic models.

### Ethical considerations

The secondary analysis presented in this study was conducted in accordance with the ethical principles outlined in the Declaration of Helsinki. The original 2018 NDHS protocol received ethical clearance from the National Health Research Ethics Committee of Nigeria (NHREC) and the Institutional Review Board of ICF International. For this specific study, ethical approval was granted by the Covenant Health Research Ethics Committee (CHREC) at Covenant University, Ota, Nigeria (protocol number: CHREC/1149/2025; NHREC registration number: NHREC/CU-HREC/1/01/2025). The research adhered to the National Code for Health Research Ethics and all relevant institutional regulations. Formal permission to access and utilize the de-identified dataset was obtained from the DHS (*Demographic and Health Surveys*) Program.

## Results

The analysis was conducted on a nationally representative sample of 28,888 married women aged 15 to 49 (design-weighted population estimate: 29,089.563). The sociodemographic profile was characterized by a majority residing in rural areas (design-weighted: 59.5%) and having received no formal or only primary education (design-weighted: 60.3%). Digital inclusion was limited, with 53.6% owning a mobile phone and 12.1% having ever used the internet. The design-weighted prevalence of the two key maternal health behaviors was low. Only 25% of women reported attending six or more antenatal care visits for their most recent birth (compared with 51% from the NDHS 2013 dataset, albeit for four or more visits ^5^). Similarly, 28.7% of women delivered their most recent baby in a health facility (compared with 36% from the NDHS 2013 survey ^30^).

The design-weighted sociodemographic characteristics of the 28,888 married women included in this analysis are presented in Table 1. The sample is primarily composed of younger women (aged 29 or below), with limited educational attainment and pronounced economic inequality. Over 60% of respondents reported no or only primary education, and almost 60% resided in rural areas. The majority of women and their partners had low educational attainment, and occupational profiles were predominantly manual or agricultural in nature. These patterns indicate persistent socioeconomic disparities that frame maternal health behavior in Nigeria. Notably, the design-weighted proportions differ from unweighted proportions for several variables, reflecting the multi-stage cluster design of the NDHS. Table 1 provides a summary of the background characteristics of the study population.

**Table 1:** Design-weighted frequency distribution of background (sociodemographic) and digital (contextual) factors among married women. 2018 *Nigeria Demographic and Health Survey* (NDHS, N = 28,888 unweighted; weighted population estimate = 29,089.563).

Variable	n (unweighted)	Unweighted (%)	Weighted (%)	SE (%)
Panel A. Background (sociodemographic) factors				
Age of the woman (years)				
29 or younger	12,112	41.9	42.5	0.5
30-39	10,092	34.9	35.3	0.4
40 or older	6,684	23.2	22.3	0.3
Highest level of education				
No education/Primary	17,535	60.7	60.3	0.8
Secondary	8,757	30.3	30.1	0.6
Higher	2,596	9.0	9.6	0.5
Wealth Index (quintile)				
Poorest	6,395	22.1	20.7	0.8
Poorer	6,267	21.7	21.4	0.8
Middle	5,853	20.3	19.3	0.6
Richer	5,531	19.1	19.2	0.7
Richest	4,842	16.8	19.4	0.7
Residence				
Rural	18,485	64.0	59.5	0.9
Urban	10,403	36.0	40.5	0.9
Geopolitical region				
North-Central	5,268	18.2	14.0	0.5
North-East	5,668	19.6	16.6	0.6
North-West	8,115	28.1	33.8	0.9
South-East	3,207	11.1	9.9	0.4
South-South	2,962	10.3	9.5	0.4
South-West	3,668	12.7	16.0	0.8
Religion				
Islam/Traditional African Religions/Other	16,626	57.6	60.3	0.9
All Christians	12,262	42.4	39.7	0.9
Number of household members				
7 or more	11,391	39.4	38.8	0.6
5 to 6	7,870	27.2	27.2	0.4
4 or less	9,627	33.3	34.0	0.5
Employment status				
Unemployed	8,624	29.9	29.5	0.5
Employed	20,264	70.1	70.5	0.5
Occupation category				
Manual/Agricultural/Other or missing	15,606	54.0	51.2	0.7
Clerical/Sales/Services	11,605	40.2	42.7	0.6
Professional/Technical/Managerial	1,677	5.8	6.1	0.3
Age of partner (years)				
29 or younger	2,957	10.2	10.1	0.3
30-39	9,298	32.2	32.7	0.4
40 or older	16,633	57.6	57.2	0.4
Highest education level of partner				
No education/Primary/Others	14,632	50.7	50.7	0.9
Secondary	9,769	33.8	33.5	0.7
Higher	4,487	15.5	15.9	0.6
Panel B. Digital (contextual) factors				
Mobile phone ownership				
No	13,674	47.3	46.4	0.7
Yes	15,214	52.7	53.6	0.7
Internet use				
Never used	25,932	89.8	87.9	0.6
Used (last 12 months or earlier)	2,956	10.2	12.1	0.6
Internet use frequency				
Not at all	26,365	91.3	89.8	0.5
At least once or more a week	2,523	8.7	10.2	0.5
Use of mobile phone for financial transactions				
No or missing	25,306	87.6	85.8	0.5
Yes	3,582	12.4	14.2	0.5

Weighted %: design-weighted percentage using NDHS individual sampling weight (V005/1,000,000); SE: standard error of the weighted percentage.

Source: National Population Commission ^3^; IBM Corporation ^29^.

Note: IBM SPSS syntax commands: CSTABULATE, /SUBPOP married women, /STATISTICS SE COUNT DEFF; weighted proportions reflect differential sampling probabilities across strata, explaining discrepancies from unweighted values.

The digital exposure profile of the same population, presented in Panel B of Table 1, reveals a pronounced digital divide. While 53.6% of women owned a mobile phone (design-weighted), only 12.1% had ever used the internet, and 10.2% used the internet at least once per week. Only 14.2% used mobile phones for financial transactions. These figures highlight that digital inclusion remains limited primarily to basic connectivity, with advanced digital engagement still relatively rare among married Nigerian women.

To explore preliminary relationships before multivariate modeling, a series of cross-tabulations was conducted between women’s background characteristics and the two primary outcomes. The results, summarized in Tables 2 and 3, present design-weighted row percentages showing the proportion of women within each predictor category who attended six or more antenatal care visits or delivered in a health facility. The formal independence test could not be computed under the current configuration because the domain variable could not be specified as a first-stage stratification variable; associations are therefore described in terms of observed patterns in design-weighted row percentages.

As shown in Table 2, antenatal care attendance was higher among women aged 30-39 (31.4%) than among those aged 29 or younger (26.3%), and was markedly lower among women aged 40 and above (12.4%). Education demonstrated a strong positive gradient: 48.8% of women with higher education and 40.3% of women with secondary education attended six or more antenatal visits, compared with only 13.6% of those with no or only primary education. Wealth showed a clear stepwise pattern, from 9.2% in the poorest quintile to 45% in the richest. Urban women were substantially more likely to attend adequate antenatal care (37.1%) than rural women (16.7%).

**Table 2:** Bivariate analysis of antenatal care attendance (design-weighted row percentages) by background (sociodemographic) factors among married women. 2018 *Nigeria Demographic and Health Survey* (NDHS).

Subcategory	Below 6 visits (%)	6 or more visits (%)	Weighted (n)
Total	75.0	25.0	29,089,563
Age of the woman (years)			
29 or younger	73.7	26.3	12,348,763
30-39	68.6	31.4	10,258,272
40 or older	87.6	12.4	6,482,528
Highest level of education			
No education/Primary	86.4	13.6	17,535,196
Secondary	59.7	40.3	8,766,564
Higher	51.2	48.8	2,787,802
Wealth Index (quintile)			
Poorest	90.8	9.2	6,007,979
Poorer	85.8	14.2	6,223,999
Middle	76.2	23.8	5,601,314
Richer	65.1	34.9	5,599,281
Richest	55.0	45.0	5,656,990
Residence			
Rural	83.3	16.7	17,299,370
Urban	62.9	37.1	11,790,193
Geopolitical region			
North-Central	77.8	22.2	4,085,729
North-East	88.8	11.2	4,841,005
North-West	87.9	12.1	9,826,362
South-East	58.8	41.2	2,893,247
South-South	62.1	37.9	2,777,481
South-West	48.8	51.2	4,665,739
Religion			
Islam/Traditional African Religions/Other	83.5	16.5	17,531,761
All Christians	62.2	37.8	11,557,802
Number of household members			
7 or more	81.6	18.4	11,282,778
5 to 6	70.1	29.9	7,906,064
4 or less	71.4	28.6	9,900,721
Employment status			
Unemployed	82.4	17.6	8,575,763
Employed	71.9	28.1	20,513,800
Occupation category			
Manual/Agricultural/Other or missing	78.5	21.5	14,898,456
Clerical/Sales/Services	73.2	26.8	12,423,334
Professional/Technical/Managerial	58.2	41.8	1,767,773
Age of partner (years)			
29 or younger	76.4	23.6	2,943,552
30-39	66.4	33.6	9,516,230
40 or older	79.7	20.3	16,629,781
Highest education level of partner			
No education/Primary/Others	86.7	13.3	14,745,091
Secondary	64.3	35.7	9,732,528
Higher	60.3	39.7	4,611,944

Weighted (n): design-weighted population size estimate.

Source: National Population Commission ^3^; IBM Corporation ^29^.

Note: IBM SPSS syntax commands: CSTABULATE with /CELLS ROWPCT, /SUBPOP married women (n = 28,888 unweighted; weighted population = 29,089.563);

Row percentages represent the proportion of women within each subcategory attending 6 or more antenatal care visits; The design-corrected independence test (Rao-Scott F-adjusted) was not available from the current analysis configuration; Associations are therefore described in terms of design-weighted row percentages.

Regional and cultural differences were pronounced. Women in the South-West recorded the highest antenatal care attendance (51.2%), followed by the South-East (41.2%), while the North-East (11.2%) and North-West (12.1%) recorded the lowest rates. Christian women showed substantially higher antenatal care utilization (37.8%) than their Muslim counterparts and those who practice Traditional African Religions (16.5%). Larger households were associated with lower antenatal care attendance (18.4% among households of seven or more members vs. 28.6% among households of four or fewer). Employed women (28.1%) were more likely to attend antenatal care than unemployed women (17.6%). Partner’s education emerged as a strong positive correlate: 39.7% of women whose partners had higher education and 35.7% of those whose partners had secondary education attended six or more antenatal care visits, compared with only 13.3% among those whose partners had little or no schooling.

Patterns for facility-based delivery (Table 3) mirrored those observed for anytenatal care attendance. Women aged 30-39 (35.6%) and those aged 29 or younger (31.2%) were more likely to deliver in a facility than those aged 40 or older (13.1%). Facility delivery rates showed a strong wealth gradient, from 8.4% in the poorest quintile to 50.5% in the richest. Urban women (42.5%) were substantially more likely to deliver in a facility than rural women (19.3%).

**Table 3:** Bivariate analysis of place of delivery (design-weighted row percentages) by background (sociodemographic) factors among married women. 2018 *Nigeria Demographic and Health Survey* (NDHS).

Subcategory	Non-health facility (%)	Health facility (%)	Weighted (n)
Total	71.3	28.7	29,089,563
Age of the woman (years)			
29 or younger	68.8	31.2	12,348,763
30-39	64.4	35.6	10,258,272
40 or older	86.9	13.1	6,482,528
Highest level of education			
No education/Primary	84.9	15.1	17,535,196
Secondary	53.2	46.8	8,766,564
Higher	42.5	57.5	2,787,802
Wealth Index (quintile)			
Poorest	91.6	8.4	6,007,979
Poorer	83.8	16.2	6,223,999
Middle	69.9	30.1	5,601,314
Richer	59.0	41.0	5,599,281
Richest	49.5	50.5	5,656,990
Residence			
Rural	80.7	19.3	17,299,370
Urban	57.5	42.5	11,790,193
Geopolitical region			
North-Central	64.8	35.2	4,085,729
North-East	80.7	19.3	4,841,005
North-West	87.9	12.1	9,826,362
South-East	45.9	54.1	2,893,247
South-South	66.6	33.4	2,777,481
South-West	50.7	49.3	4,665,739
Religion			
Islam/Traditional African Religions/Other	81.0	19.0	17,531,761
All Christians	56.6	43.4	11,557,802
Number of household members			
7 or more	77.8	22.2	11,282,778
5 to 6	66.3	33.7	7,906,064
4 or less	67.8	32.2	9,900,721
Employment status			
Unemployed	78.2	21.8	8,575,763
Employed	68.4	31.6	20,513,800
Occupation category			
Manual/Agricultural/Other or missing	74.0	26.0	14,898,456
Clerical/Sales/Services	70.6	29.4	12,423,334
Professional/Technical/Managerial	53.5	46.5	1,767,773
Age of partner (years)			
29 or younger	72.9	27.1	2,943,552
30-39	60.7	39.3	9,516,230
40 or older	77.0	23.0	16,629,781
Highest education level of partner			
No education/Primary/Others	85.5	14.5	14,745,091
Secondary	59.0	41.0	9,732,528
Higher	51.6	48.4	4,611,944

Weighted (n): design-weighted population size estimate.

Source: National Population Commission ^3^; IBM Corporation ^29^.

Note: IBM SPSS syntax commands: CSTABULATE with /CELLS ROWPCT, /SUBPOP married women (n = 28,888 unweighted; weighted population = 29,089.563);

Row percentages represent the proportion of women within each subcategory delivering in a health facility;

The design-corrected independence test was not available from the current analysis configuration.

Regional disparities were equally pronounced. The South-East recorded the highest facility delivery rate (54.1%) and the North-West the lowest (12.1%). The North-Central region (35.2%) showed considerably higher facility delivery than the South-South (33.4%), despite comparable antenatal care rates. Christian women had higher facility delivery rates (43.4%) than their Muslim counterparts and those who practice Traditional African Religions (19%). Women in smaller households were more likely to deliver in a facility, and employed women (31.6%) were more likely to deliver in a facility than those who were unemployed (21.8%). Partner’s age was also associated with facility delivery: women whose partners were aged 30-39 showed the highest facility delivery rate (39.3%), compared with 27.1% for partners aged 29 or younger and 23% for partners aged 40 or older. The education level of the partner remained a strong positive correlate: 41% of women whose partners had secondary education and 48.4% of those with partners with higher education delivered in a facility, compared with 14.5% among those whose partners had little or no schooling.

The bivariate analysis (Table 4) shows patterns of association between digital access indicators and attendance at six or more antenatal care visits among married women. At the bivariate level, all forms of digital engagement were positively associated with adequate antenatal care attendance. Women who owned a mobile phone were substantially more likely to complete six or more antenatal care visits (34.9%) than non-owners (13.5%). Women who had ever used the internet showed markedly higher antenatal care attendance rates (50.9%) than those who had never used the internet (21.4%). Women who used the internet at least once per week also showed higher antenatal care attendance (49.1%) than those who reported no internet use (22.3%). Women who used mobile phones for financial transactions had notably higher rates of adequate antenatal care attendance (45.7%) compared with those who did not (21.6%). As discussed in the multivariate analysis (Table 5), the direction of some of these associations reverses after adjustment for socioeconomic confounders.

**Table 4:** Bivariate analysis of digital (contextual) exposure (design-weighted row percentages) by antenatal care attendance and place of delivery among married women. 2018 *Nigeria Demographic and Health Survey* (NDHS).

Subcategory	Antenatal care attendance (%)		Place of delivery (%)		Weighted (n)
	Below 6 visits	6 or more visits	Non-facility	Facility	
Total	75.0	25.0	71.3	28.7	29,089,563
Mobile phone ownership					
No	86.5	13.5	84.9	15.1	13,492,308
Yes	65.1	34.9	59.5	40.5	15,597,255
Internet use					
Never used	78.6	21.4	75.2	24.8	25,567,913
Used (last 12 months or earlier)	49.1	50.9	43.0	57.0	3,521,651
Internet use frequency					
Not at all	77.7	22.3	74.5	25.5	26,131,252
At least once or more a week	50.9	49.1	42.7	57.3	2,958,311
Use of mobile phone for financial transactions					
No or missing	78.4	21.6	74.9	25.1	24,969,754
Yes	54.3	45.7	49.3	50.7	4,119,809

Source: National Population Commission ^3^; IBM Corporation ^29^.

Note: IBM SPSS syntax commands: CSTABULATE with /CELLS ROWPCT, /SUBPOP married women (N = 28,888 unweighted; weighted population = 29,089.563).

Antenatal care attendance: proportion of women within each digital-exposure category attending 6 or more antenatal care visits for their most recent birth. Place of delivery: proportion of women within each digital-exposure category delivering their most recent baby in a health facility;

Weighted (n): design-weighted population size; identical for antenatal care and place of delivery analyses because the denominator is the full married women subpopulation;

At the bivariate level, all digital-exposure variables showed positive associations with both outcomes; the negative independent associations for internet use frequency (antenatal care) and mobile financial transactions (place of delivery) emerge only in the multivariate model (Table 5) after adjustment for socioeconomic confounders;

The design-corrected independence test was not available from the current analysis configuration.

**Table 5:** Multivariate Complex Samples binary logistic regression of antenatal care attendance and place of delivery among married women in Nigeria. 2018 *Nigeria Demographic and Health Survey* (NDHS).

Predictor	Antenatal care (6 or more visits)		Place of delivery (facility)	
	OR	95%CI	OR	95%CI
Background (sociodemographic) factors				
Age of the Woman (reference: 29 or younger)				
30-39	0.911	0.823-1.009	0.901	0.821-0.990
40 or older	0.288	0.249-0.333	0.248	0.216-0.284
Highest level of education (reference: no education/Primary)				
Secondary	1.444	1.292-1.614	1.661	1.479-1.865
Higher	2.002	1.642-2.442	2.400	1.948-2.956
Wealth Index - quintile (reference: poorest)				
Poorer	1.225	1.046-1.435	1.641	1.399-1.925
Middle	1.418	1.196-1.681	2.357	1.984-2.800
Richer	1.495	1.246-1.793	2.476	2.053-2.986
Richest	1.546	1.258-1.899	2.654	2.167-3.249
Residence (reference: rural)				
Urban	1.246	1.122-1.385	1.264	1.130-1.413
Geopolitical region (reference: North-Central)				
North-East	0.611	0.508-0.735	0.679	0.566-0.816
North-West	0.712	0.598-0.848	0.388	0.328-0.459
South-East	1.924	1.620-2.285	1.529	1.302-1.796
South-South	1.672	1.410-1.983	0.546	0.456-0.654
South-West	2.976	2.570-3.445	1.206	1.030-1.412
Religion (reference: Islam/Traditional African Religions/Other)				
All Christians	1.163	1.001-1.351	1.293	1.123-1.489
Number of household members (reference: 7 or more)				
5 to 6	0.902	0.800-1.016	0.844	0.759-0.939
4 or less	0.657	0.598-0.723	0.606	0.549-0.669
Employment status (reference: unemployed)				
Employed	1.218	1.098-1.351	1.151	1.036-1.280
Occupation category (reference: manual, agricultural, other, or missing)				
Clerical/Sales/Services	1.022	0.922-1.133	0.991	0.904-1.086
Professional/Technical/Managerial	0.877	0.713-1.078	0.793	0.655-0.960
Age of partner (reference: 29 or younger)				
30-39	1.129	0.995-1.280	1.199	1.054-1.363
40 or older	0.821	0.700-0.964	0.822	0.715-0.946
Highest education of partner (reference: no education, primary, others)				
Secondary	1.364	1.222-1.523	1.487	1.333-1.659
Higher	1.488	1.304-1.698	1.798	1.569-2.060
Digital (contextual) factors				
Mobile phone ownership (reference: no)				
Yes	1.290	1.164-1.430	1.357	1.239-1.486
Internet use (reference: Never used)				
Used (last 12 months or earlier)	1.638	1.295-2.073	1.082	0.843-1.388
Internet use frequency (reference: not at all)				
At least once a week	0.599	0.470-0.764	1.022	0.775-1.349
Use of mobile phone for financial transactions (reference: no or missing)				
Yes	0.884	0.781-1.000	0.781	0.689-0.887

95%CI: 95% confidence interval; OR: odds ratio.

Antenatal care model classification accuracy: 79.2% overall (otherwise, or others *−* ELSE: 92.2%; 6 or more visits: 40.2%). Place of delivery model classification accuracy: 77.7% overall (ELSE: 89.2%; health facility: 49.3%).

Reference category for both outcomes: ELSE/non-event (code 1). Subpopulation: married or cohabiting women (N = 28,888 unweighted; weighted population = 29,089.563). Design df = 1,315.

OR from CSLOGISTIC /ODDSRATIOS FACTOR = [var(1.00)] with /TEST TYPE = ADJF (IBM SPSS syntax commands).

Similar patterns were observed between digital exposure and the likelihood of delivering in a health facility (Table 4). At the bivariate level, all digital engagement indicators were positively associated with facility delivery. Women who owned a mobile phone were substantially more likely to deliver in a facility (40.5%) than non-owners (15.1%). Internet users showed the highest facility delivery rates (57%) compared with those who had never used the internet (24.8%). Women who used the internet frequently (57.3%) and those who used mobile phones for financial transactions (50.7%) also showed substantially higher facility delivery rates than their counterparts (25.5% and 25.1%, respectively). The multivariate model, which adjusts for socioeconomic confounders, reveals a more nuanced picture.

Collectively, these bivariate results confirm that both maternal and partner’s education, household wealth, employment, religion, and place of residence are important factors that shape maternal health behavior in Nigeria. These associations provide an essential contextual foundation for interpreting the multivariate results that follow.

The results of the comprehensive multivariate Complex Samples binary logistic regression model are presented in Table 5. The antenatal care model correctly classified 79.2% of cases (otherwise, or others *−* ELSE: 92.2%; 6 or more: 40.2%), and the place of delivery model correctly classified 77.7% of cases (ELSE: 89.2%; facility: 49.3%).

As shown in Table 5, traditional sociodemographic and economic factors are the most powerful predictors. A strong graded relationship was observed for both education and wealth. Women with higher education had 2.40 times the OR of delivering in a health facility (OR = 2.40, 95%CI: 1.95-2.96, p < 0.001) compared with women with no or primary education, and 2.00 times the odds of adequate antenatal care attendance (OR = 2.00, 95%CI: 1.64-2.44, p < 0.001). Women in the richest wealth quintile had 2.65 times the odds of a facility-based delivery (OR = 2.65, 95%CI: 2.17-3.25, p < 0.001) compared with those in the poorest quintile. Geographic disparities were profound. A markedly divergent pattern was observed in the South-South region, where women had significantly higher odds of adequate antenatal care attendance (OR = 1.67, 95%CI: 1.41-1.98, p < 0.001) but significantly lower odds of delivering in a health facility (OR = 0.55, 95%CI: 0.46-0.65, p < 0.001) compared with the North-Central reference region.

In the comprehensive model, digital engagement variables emerged as significant independent predictors alongside these powerful sociodemographic drivers. Mobile phone ownership was consistently and positively associated with both outcomes, increasing the odds of adequate antenatal care attendance by 29% (OR = 1.29, 95%CI: 1.16-1.43, p < 0.001) and facility delivery by 35.7% (OR = 1.36, 95%CI: 1.24-1.49, p < 0.001). Other forms of digital engagement revealed a more complex relationship. Having ever used the internet was associated with significantly higher odds of adequate antenatal care attendance (OR = 1.64, 95%CI: 1.30-2.07, p < 0.001), but was not independently associated with facility delivery (OR = 1.08, 95%CI: 0.84-1.39, p > 0.05). By contrast, frequent internet use (at least once per week) was associated with significantly lower odds of adequate antenatal care attendance (OR = 0.60, 95%CI: 0.47-0.76, p < 0.001), though it was not independently associated with facility delivery (OR = 1.02, 95%CI: 0.78-1.35, p > 0.05). Use of a mobile phone for financial transactions showed a borderline association with antenatal (OR = 0.88, 95%CI: 0.78-1.00) and a significant negative association with facility delivery (OR = 0.78, 95%CI: 0.69-0.89, p < 0.001).

## Discussion

This study developed an integrated model to explore the complex interplay of sociodemographic and digital factors influencing maternal health behaviors in Nigeria. The findings confirm that while traditional determinants, such as education and wealth, remain the most powerful drivers, the digital landscape introduces a new layer of complexity that is not uniformly positive.

The overwhelming influence of education and wealth reaffirms the foundational principles of the social determinants of health framework. These factors equip women with the financial resources, health literacy, and autonomy necessary to overcome barriers to care. A key finding is the consistent, positive, and independent effect of basic mobile phone ownership on both outcomes. This suggests that even the simplest form of digital access serves as a powerful tool, likely by reducing informational barriers and facilitating communication. This aligns with the health belief model, where a mobile phone can act as a persistent “cue to action”.

A further important finding is the significant negative association between maternal age (40 or older) and service utilization. Women in this age group had 71.2% lower odds of attending six or more antenatal care visits (OR = 0.29) and 75.2% lower odds of delivering in a health facility (OR = 0.25) compared with the youngest cohort. Women aged 30-39 did not differ significantly from the youngest group for antenatal care attendance, suggesting the age penalty is concentrated in the oldest reproductive age group. This is an important policy insight: older, higher-parity women may rely more on previous birth experience and represent a key underserved group requiring targeted outreach.

The geographic divergence in the South-South region is a particularly important policy finding. Women there had significantly higher odds of adequate antenatal care attendance (OR = 1.67) yet significantly lower odds of facility delivery (OR = 0.55), both compared with the North-Central reference region. This divergence suggests that the decision to attend routine antenatal care and the decision about where to give birth are influenced by distinct and partially independent factors. This may point to specific, localized barriers to facility-based delivery in the South-South, such as poor quality of intrapartum care or prohibitive informal costs.

The most novel and important findings emerge from examining more advanced forms of digital engagement. A critical distinction must be drawn between two internet-related predictors that operate in opposing directions. Internet access (having ever used the internet) was independently and positively associated with antenatal care attendance at the multivariate level (OR = 1.64, p < 0.001), indicating that basic internet access increases the likelihood of women seeking adequate antenatal care. However, frequent internet use (at least once per week) was independently and negatively associated with antenatal care attendance (OR = 0.60, p < 0.001) after controlling for all sociodemographic factors. Both variables were non-significant for facility delivery. This divergence within a single domain of digital engagement is a novel finding. A plausible interpretation is that basic internet access acts as an informational gateway that prompts women to seek formal services. However, among frequent internet users, after controlling for the strong socioeconomic advantages that correlate with frequent use, the residual relationship between frequency and antenatal care attendance is negative, potentially reflecting a subset of digitally engaged women who use online channels to seek alternative health information or to access private or informal services outside the measured formal antenatal care pathway.

Mobile phone use for financial transactions showed a borderline association with antenatal care (OR = 0.88, 95%CI: 0.78-1.00) and a significant negative independent association with facility delivery (OR = 0.78, 95%CI: 0.69-0.89) in the multivariate model. This negative association, which emerges only after adjusting for socioeconomic status, is a paradox that warrants further investigation. Notably, at the bivariate level, financial transaction users showed substantially higher rates of both antenatal care attendance (45.7% vs. 21.6%) and facility delivery (50.7% vs. 25.1%). The negative adjusted OR suggests that, once socioeconomic confounders are controlled, women who use mobile financial services may be choosing alternative birth settings, such as private facilities or community-based attendants who now accept mobile payments, rather than the formal public facilities captured in this measure.

Finally, the divergent regional patterns, especially in the South-South zone, highlight that maternal health behavior is not a monolithic concept. The decision to attend routine check-ups is different from the high-stakes decision of where to give birth. This divergence likely points to specific, localized barriers to facility-based delivery, such as poor quality of intrapartum care or prohibitive informal costs.

The strong predictive power of education and wealth in the model is highly consistent with extensive research in Nigeria ^1^ ^,^ ^5^. Similarly, the positive impact of basic mobile phone ownership aligns with other studies ^4^ ^,^ ^25^. The nuanced findings related to advanced digital use add a critical new dimension to the literature. While many studies focus on the barriers to mHealth adoption among the digitally excluded, this study reveals unexpected patterns among the digitally included, complicating the narrative of digital technology as a simple tool for funneling users into the formal health system.

The implications of this integrated model are significant. First, technology is an accelerator, not a substitute for equity. Long-term investments in girls’ education and women’s economic empowerment are fundamental maternal health policies. Second, policymakers must not assume that digital inclusion will automatically translate into higher utilization of public health facilities. It is essential to investigate why empowered women may be opting out and to address underlying issues of quality, cost, and patient experience. Third, interventions must be tailored to specific behaviors and regions. Finally, the strong, positive effect of simple mobile phone ownership confirms that scaling up proven, low-cost interventions, such as SMS reminders, is a high-impact strategy.

The primary strengths of this study lie in its use of a large, nationally representative dataset and in the development of a comprehensive model. However, the cross-sectional design precludes the establishment of causality. The data are from 2018, and the digital landscape has evolved since then. Furthermore, the survey’s variables for digital engagement are relatively basic. Qualitative studies are needed to explore the motivations behind the counterintuitive findings. Longitudinal research is also required to track how women’s health behaviors evolve as their digital skills grow.

Furthermore, the model did not explore the potential causal or mediational pathways between variables. Sociodemographic factors, such as education and wealth, undoubtedly influence access to digital resources. Future research could employ methods such as hierarchical regression or structural equation modeling to explicitly test the mediating role that digital engagement plays in the relationship between structural inequities and maternal health behaviors.

A primary limitation of this study is the use of data from 2018. The digital landscape in Nigeria has undergone exponential evolution since the survey was conducted. The 2018 design-weighted estimates showed 53.6% mobile phone ownership and 10.2% weekly internet use among married women. However, by early 2024, the Nigerian Communications Commission (NCC) reported over 210 million active mobile subscriptions and 160 million active internet subscriptions ^31^. This rapid digital increase does not diminish the findings; rather, it significantly heightens their relevance. A further methodological limitation is that the design-corrected independence test could not be computed because the domain variable could not be specified as a first-stage stratification variable.

The findings suggest that as digital inclusion becomes nearly universal, understanding how women use these tools, rather than just whether they have access to them, is an essential question for maternal health policy.

It is essential to reiterate that these findings are, by design, based solely on the 28,888 married or cohabiting women in the sample. This study does not capture the experiences of unmarried women. This population faces a different set of social and structural barriers, and their health-seeking behavior in the digital age constitutes a distinct, though equally important, area for future research.

## Conclusion

In summary, the results of this comprehensive modeling effort substantiate the hypothesis that while the role of traditional socioeconomic determinants in shaping maternal health behaviors remains powerful and entrenched, digital factors exert a significant and independent influence, the effects of which are confirmed to be complex and nonlinear. While education and wealth are powerful determinants, these findings show that the role of technology is not straightforward. Basic mobile phone ownership consistently acts as a bridge to services. However, a more nuanced picture emerges for advanced digital engagement: internet access is independently and positively associated with antenatal care attendance, while frequent internet use is independently and negatively associated, revealing two distinct pathways operating within the same digital domain. Similarly, mobile financial transactions are negatively associated with facility delivery after controlling for socioeconomic factors. These paradoxes suggest that as women become more digitally and financially autonomous, they may be empowered to make alternative choices within health care. These findings have profound implications for policy. To be effective, interventions must be multisectoral, addressing the foundational barriers of poverty and educational disadvantage while leveraging the power of digital tools. Simply promoting digital access is insufficient; we must also understand the diverse and evolving ways in which empowered women use technology to navigate their health journeys.

## Data Availability

The sources of information used in the study are indicated in the body of the article.
